# Dopamine-mediated improvements of the step threshold task in Parkinson’s disease: validation against clinical measures of motor and cognitive function

**DOI:** 10.3389/fnhum.2025.1648250

**Published:** 2025-11-13

**Authors:** Alyson N. Moll, Harrison C. Walker, Noah Rosenblatt, Daniel J. Kuhman, Jaden Adams, Victor A. Del Bene, Roy C. Martin, Sarah Brinkerhoff, Christopher P. Hurt

**Affiliations:** 1 Rehabilitation Science, University of Alabama at Birmingham, Birmingham, AL, United States; 2 Department of Neurology, University of Alabama at Birmingham, Birmingham, AL, United States; 3 Center for Lower Extremity Ambulatory Research, Rosalind Franklin University of Medicine and Science, North Chicago, IL, United States; 4 Department of Physical Therapy, University of Alabama at Birmingham, Birmingham, AL, United States

**Keywords:** Parkinson’s disease, balance, step threshold, postural instability, validation

## Abstract

**Introduction:**

Standardized treadmill-based balance disturbances have potential to improve assessments of dynamic balance control in individuals with Parkinson’s disease. Here we examined the validity of a step threshold task to measure dynamic balance control in patients with Parkinson’s disease.

**Methods:**

Thirty-nine participants with idiopathic Parkinson’s disease underwent clinical testing and performed a dynamic balance assessment both OFF and ON dopaminergic medication. For the assessment, participants were instructed to avoid stepping in response to progressively larger postural perturbations applied via treadmill translations. The step threshold was defined as the perturbation magnitude that resulted in a stepping response on four consecutive trials. Validity was assessed by correlating medication-mediated changes in gold standard clinical measures and medication-mediated changes in stepping.

**Results:**

Medication-mediated changes in step threshold correlated with changes in MDS-UPDRS part III (*p* < 0.01), with change in MDS-UPDRS 3.12 postural instability (*p* < 0.05), and with measures of executive function: CPT-3 Omission T-score (*p* = 0.013), the CPT-3 Commission T-score (*p* = 0.019), and the CPT-3 Variability T-score (*p* = 0.040).

**Discussion:**

Our results validate step threshold task as a measure of dynamic balance control in patients with Parkinson’s disease. Correlations with gold standard assessments of motor and executive function suggest that the step threshold task can serve as a comprehensive measure of dynamic balance control.

## 1 Introduction

Parkinson’s disease (PD) is a neurological disorder that negatively impacts motor and cognitive function, resulting in decreased balance control (Horak, 1997; Horak et al., 1997; Allen et al., 2013; Dirnberger and Jahanshahi, 2013; Nyatega et al., 2022; Çekok et al., 2024). Quantitative assessments of balance control are important because they serve as key outcome variables to assess interventions, evaluate treatment efficacy, and monitor disease progression. While many clinical measures can be used to assess balance control (Viveiro et al., 2019; Lu et al., 2023; Jansen et al., 2025), these measures have limitations. As an example, the retropulsion or pull test (Nonnekes et al., 2015), a gold standard test, has some subjective elements in execution and rating that increase variation within and between participant responses (Jansen et al., 2025). Further many clinical balance assessments are subject to ceiling effects (Lewis et al., 2020) and significant declines in performance may be required before reaching a clinically important threshold (Lewis et al., 2020). Thus, new standardized measures that overcome these limitations are needed.

Recent studies show that treadmill perturbation protocols have the potential to improve balance assessments by standardizing test delivery to individuals with PD compared to more coarse clinical measures (Kuhman et al., 2020; Lu et al., 2023; Jansen et al., 2025). However, these standardized perturbation protocols have yet to be validated in this population. Valid balance assessments for patients with PD should differentiate aspects of function that are known to be affected by medication or disease processes. Previous research on the impact of medication on balance performance is equivocal (Bloem et al., 1996; Di Giulio et al., 2016; Kuhman et al., 2020). Recently, using a novel balance perturbation protocol, we demonstrate that dynamic balance control improves with dopaminergic medications, suggesting that the step threshold task paradigm captures the impact of dopamine deficiency (and its replacement) on balance control (Kuhman et al., 2020).

Here we used a step threshold protocol to determine the perturbation magnitude that yielded a compensatory step when the participant was instructed to actively resist taking the step (Goetz et al., 2008; Merello et al., 2011). The protective stepping response is a natural reaction, and in absence of instructions, is often utilized even though an individual is dynamically stable (Mille et al., 2003). Discouraging the stepping response during perturbation (Kuhman et al., 2020) probes inhibitory control, in which poor inhibition response has been related to worse dynamic balance control by individuals with PD (Xu et al., 2014; Çekok et al., 2024). Thus, our paradigm examines executive function in addition to mechanical processes necessary to recover balance. We validate this protocol using gold standard assessments of balance control and executive function.

## 2 Materials and methods

### 2.1 Participants

Thirty-nine individuals diagnosed with idiopathic PD were included in this institutionally approved study (Table 1) (mean age: 63 years ± 8, UPDRS OFF medication: 49 ± 13) as part of a clinical trial, the SUNDIAL Trial, a longitudinal deep brain stimulation (DBS) study (clinicaltrials.gov NCT03353688). All assessments were completed prior to DBS surgery. Participants in this study were approached after recommendation from a multidisciplinary Movement Disorders committee for unilateral subthalamic nucleus DBS as part of routine care. Inclusion required ≥ 30% improvement in the Movement Disorder Society-Sponsored Revision of the Unified Parkinson’s Disease Rating Scale (MDS-UPDRS) part III on dopaminergic medications versus the practically defined “OFF” state (≥ 12 h OFF medications), Hoehn and Yahr score > 1, and Dementia Rating Scale-2 score ≥ 130. Exclusion criteria included duration of disease < 4 years, and no history of stroke or other significant neurological conditions. To evaluate global cognition in Parkinson’s disease, all participants completed the Dementia Rating Scale–Second Edition (DRS-2), which has been found to be a valid global cognitive screener in PD (Lopez et al., 2023). This was included to ensure PD patients with dementia were not included in the DBS trial.

**TABLE 1:** Participant characteristics.

Characteristics	Sex	Race	Age (years)	PD onset (years)	FOGQ	ABC	Falls	DRS-2 score	LEDD (mg)
$\bar{\text{x}}$	27 m	35C 2 A 2AA	58.6	50.4	6.3	78.2	46.2%	138.2	976.6
SD			7.6	7.9	4.9	17.5		3.2	476.3

$\bar{\text{x}}$, mean; m, male; C, Caucasian; DRS-2, Dementia Rating Scale–Second Edition Total Score; AA, African American; A, Asian.

### 2.2 Experimental protocol

Participants performed the step threshold task and the MDS-UPDRS part III motor exam (Goetz et al., 2008; Merello et al., 2011). Higher scores on the step threshold indicated greater balance control, while higher scores on the MDS-UPDRS part III indicated a greater effect of PD on fine and or gross motor function (i.e., greater disease severity). Initially tasks were performed in the OFF-medication state, and then the ON medication state (∼1 h after self-administering medication). Confirmation of the ON medication state was a combination of observation by the research team, participant report, and symptom reduction. All participants completed the Freezing of Gait Questionnaire (FOGQ), where higher scores also represent greater disease severity.

**FIGURE 1:**
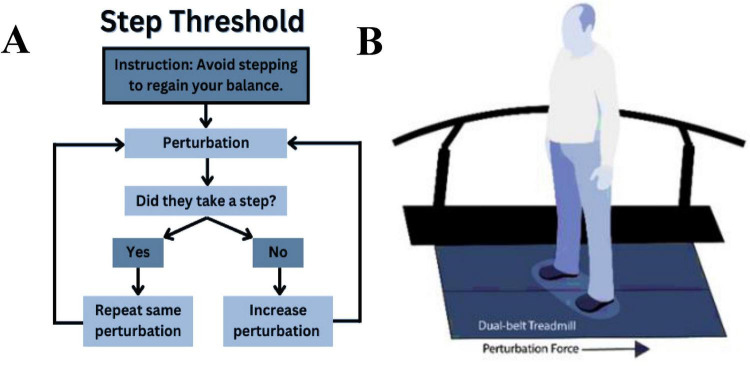
Instructions given to the participants performing the step threshold task **(A)** and position of the participant standing on the treadmill about to perform the step threshold task **(B)**.

To identify step thresholds, participants stood on a force-instrumented treadmill (Motek, Amsterdam, Netherlands) and experienced progressively increasing backward-directed perturbations (Jacobs and Horak, 2007; Kuhman et al., 2020). Treadmill perturbations consisted of a 400 ms (200 ms acceleration and 200 ms deceleration) triangular acceleration wave (Kuhman et al., 2020) controlled using custom software in D-Flow (Motek, Amsterdam, Netherlands), with the start of the perturbation randomized to occur 3–6 s after the trial was initiated. We instructed participants to “avoid taking a step to these postural perturbations unless you must” (Figure 1A). If a participant stepped, the perturbation magnitude was repeated up to three more times; the inability to avoid a step on four consecutive trials at a given magnitude defined the step threshold (Crenshaw and Kaufman, 2014). The repeated attempts provided participants an opportunity to modify their perturbation response (Kuhman et al., 2020), which in older adults, happens within 2–3 exposures of the same perturbation (Owings et al., 2001). If the participant did not step at a given magnitude, we increased the treadmill belt acceleration by 0.5 m/s^2^ on the subsequent trial (Figure 1B).

Participants completed neuropsychological testing on a contiguous day from when they performed the step threshold task. To avoid motor symptoms interfering in the neuropsychological assessment, all individuals were ON their PD medications. Assessments included the Conners’ Continuous Performance Test (CPT-3) (v3, Multi-Health System), which is a widely used neuropsychological test of sustained attention and response inhibition (Go/No-Go paradigm) and is highly sensitive to changes in cognitive function (Riccio et al., 2002). Participants were asked to quickly hit the space bar to all letters except the letter “X,” when they were instructed to inhibit their response. The test took 14 min to complete and comprised of 360 trials, with the inter-stimulus interval ranging 1–4 s. The CPT-3 has several possible outcomes related to attention and impulsivity/inhibition. *A priori*, we selected several CPT-3 outcome scores related to response inhibition and within-person reaction time consistency (Omissions, Commissions, Variability). All scores were age-corrected and higher T-scores reflect worse performance (i.e., greater inattention, worse response inhibition, or increased within-person variability in reaction times). Due to the COVID-19 pandemic, CPT-3 was only collected in a subset (i.e., 26) of the 39 individuals.

### 2.3 Statistical analyses

To establish construct validity, we assessed known groups validity, convergent validity and discriminant validity. For known groups validity, we used paired samples *t*-test to compare step thresholds in the ON vs. OFF condition. While some aspects of gait and balance do not respond as well to levodopa, we and others have shown that medication improves recovery from perturbations (Horak et al., 1997; Bronte-Stewart et al., 2002; Kuhman et al., 2020). To assess convergent validity, we used Pearson correlation to quantify associations between ON-OFF changes in stepping thresholds with those of MDS-UPDRS part III, as well as with those of item 3.12, which rates individuals’ ability to recover from a backward pull (retropulsion test). We also correlated changes in stepping thresholds to the postural instability and gait dysfunction (PIGD) subscale (Jankovic and Kapadia, 2001) of the MDS-UPDRS part III using a Pearson correlation. We assessed discriminant validity by correlating medication-mediated changes in step threshold to tremor score. If medication-induced changes in stepping threshold reflect changes in dynamic balance control, then they should be independent of changes to tremor score because they are different constructs. We assessed criterion validity by correlating stepping thresholds to executive function (Xu et al., 2014). To explore the relationship between stepping threshold and executive functioning, we correlated thresholds with previously described subscales within CPT-3 using Spearman rho statistic (Nixon et al., 2010). Data samples < 30 (due to the COVID pandemic, we only performed the neuropsychological test on 26 participants) are often considered skewed based on the central limit theorem (Nixon et al., 2010) and require non-parametric statistics. For all correlational measures, coefficients of 0.60 and above are considered excellent, 0.30–0.59 are considered adequate and < 0.30 are considered poor (Andresen, 2000). We also wanted to determine the minimal detectable change (MDC) in the step threshold given our relatively large sample in the current study. All statistical analyses were conducted using SPSS and an alpha level of 0.05 was used to detect statistical significance.

## 3 Results

### 3.1 Participant characteristics and medication-mediated effects on function

Demographic data for participants in this study are presented in Table 1. Forty-six percent of patients with PD reported a fall in the past 12 months. In addition, scores for FOGQ, ABC, and DRS-2 scores are reported in Table 1. Clinical scores showed an improvement for overall MDS-UPDRS total (OFF: 81.7 ± 23.0, ON: 59.6 ± 19.2, *p* < 0.001) (Table 2); MDS-UPDRS part III (OFF: 48.5 ± 13.1, ON: 26.3 ± 9.7, *p* < 0.001) (Table 2); and MDS-UPDRS 3.12 postural instability (OFF: 0.7 ± 1.0, ON: 0.1 ± 0.5, *p* < 0.001) (Table 2). The MDC value of step threshold that reflects a true change in task performance is estimated to be 0.46 ms^–2^.

**TABLE 2:** Participant scores from clinical test (MDS-UPDRS).

	MDS-UPDRS III score		PIGD score		3.12 PI score	
Med status	OFF meds	ON meds	OFF meds	ON meds	OFF meds	ON meds
Mean	48.5	26.3	0.8	0.5	0.7	0.1
SD	13.1	9.7	0.5	0.4	1.0	0.5

### 3.2 Step threshold and clinical measures

**FIGURE 2:**
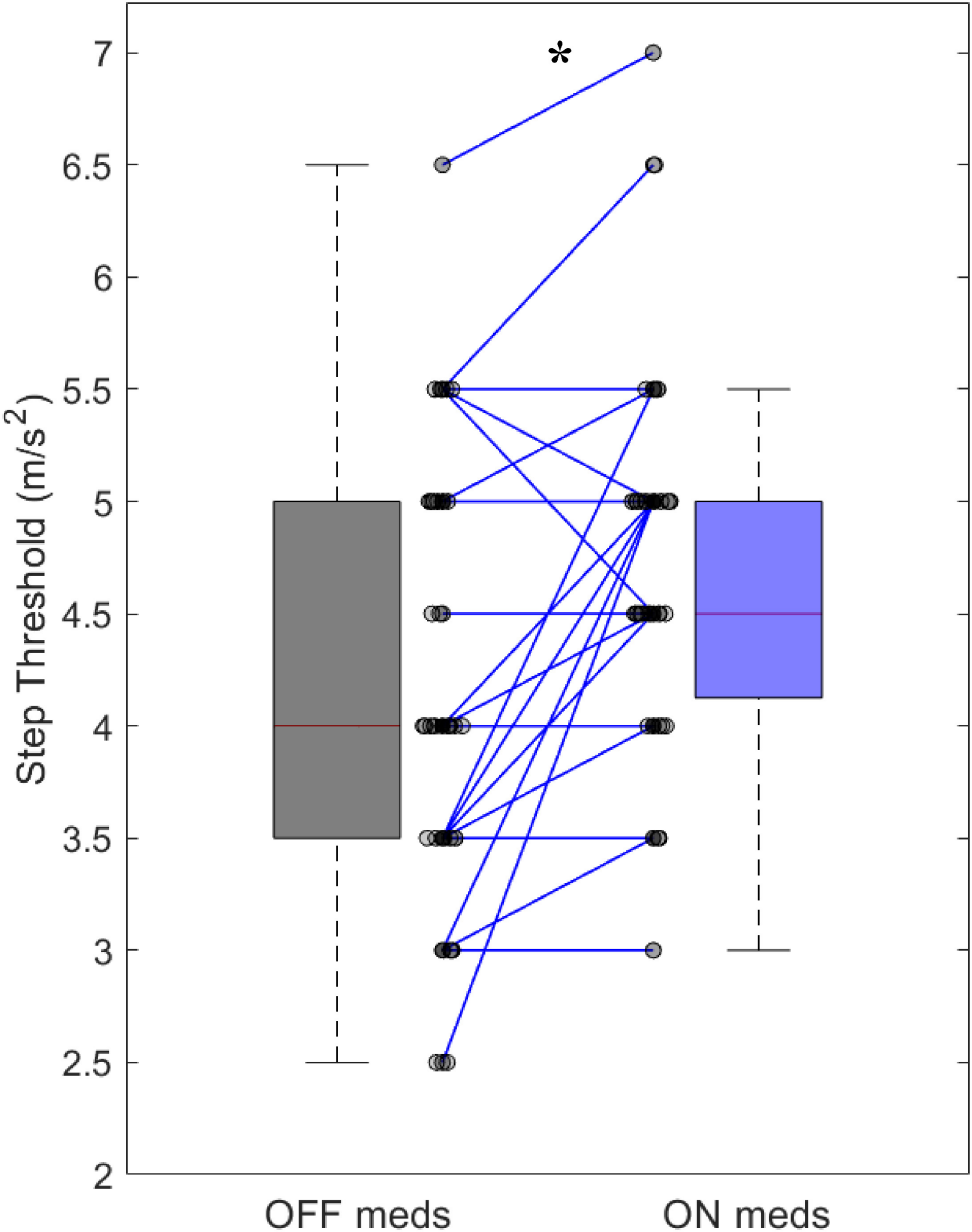
Step threshold difference between OFF and ON medications of 39 participants (*p < 0.001).

Improvements in clinical measures of motor function related to our step threshold measure. Step thresholds increased with dopaminergic medications (OFF: 4.15 ± 0.92 ms^–2^, ON: 4.69 ± 0.77 ms^–2^, *p* < 0.001) (Figure 2). The medication-mediated change in step threshold correlated to the change in MDS-UPDRS part III (*r* = –0.47, *p* < 0.01) (Table 3) and to the change in MDS-UPDRS 3.12 postural instability subscore (*r* = –0.35, *p* < 0.05) (Table 3). The medication-mediated change in step threshold did not significantly correlate to the change in PIGD (*r* = –0.03, *p* > 0.05) (Table 3). The association between the change in step threshold and a change in the tremor score was not significant (*r* = –0.27, *p* > 0.05).

**TABLE 3:** Correlations between stepping thresholds (ST) clinical measures.

Step threshold	MDS-UPDRS III (OFF)	MDS-UPDRS III (ON)	MDS-UPDRS III (Δ)	PIGD (OFF)	PIGD (ON)	PIGD (Δ)	3.12 (OFF)	3.12 (ON)	3.12 (Δ)
ST(OFF)	–0.19	–0.08	–0.34*	–0.25	–0.22	–0.13	–0.08	0.01	–0.09
ST(ON)	0.28	0.32*	0.06	0.03	0.15	–0.13	0.22	–0.03	0.24
ST(Δ)	–0.51^#^	–0.23	–0.47^#^	–0.33*	–0.41^#^	–0.03	–0.32*	0.05	–0.35*

*N* = 39;

*p < 0.05;

^#^*p* < 0.01 (correlation coefficient).

### 3.3 Step threshold and neuropsychological testing

Step threshold scores for participants OFF or ON dopaminergic medication were significantly correlated with measures from CPT-3 testing while participants were ON dopaminergic medication. Step threshold OFF medication correlated with all three CPT-3 outcomes (*p* < 0.04 for all comparisons) (Table 4). The correlation between step threshold and CPT-3 perseverance T-score was not statistically significant (*p* = 0.06) (Table 4). The difference in step threshold with medication (ON vs. OFF) was significantly correlated to CPT-3 Omission T-score), CPT-3 perseverance and the CPT-3 Variability score (*p* < 0.03 for all) (Table 4). The correlation between step threshold and CPT-3 Commission T-score was not statistically significant (*p* = 0.06) (Table 4).

**TABLE 4:** ST and neuropsychological measures.

Step threshold	CPT3 omission (ON)	CPT3 perseverance (ON)	CPT3 commissions (ON)	CPT3 variability (ON)
ST(OFF)	–0.48*	–0.27	–0.46*	–0.41*
ST(ON)	0.27	0.40*	–0.15	0.15
ST(Δ)	0.59^#^	0.46*	0.33	0.43*

*N* = 26;

*p < 0.05;

^#^*p* < 0.01 (correlation coefficient).

## 4 Discussion

Our results validate the step threshold task for assessing dynamic balance in individuals with PD. First, in a group of 39 individuals with PD, step threshold significantly improved with dopaminergic medications demonstrating known groups validity. Second, improvements in step threshold with medication correlated with medication-related improvements in the MDS-UPDRS 3.12 scores, a clinical test of responsiveness to postural perturbation (Goetz et al., 2008), as well as improvements in the MDS-UPDRS part III a test of motor function in PD (Goetz et al., 2008), demonstrating convergent validity. Third, we also showed that improvements in step threshold did not correlate to the tremor subscale of the MDS-UPDRS III, demonstrating discriminant validity. Finally, the step threshold OFF and the change in step threshold correlated with measures of executive function demonstrating convergent validity. Results showed adequate correlations for a validation study. This finding suggests that improvements in dynamic balance control may result from improved motor and executive function (Dirnberger and Jahanshahi, 2013).

The step threshold assessment employs progressively larger treadmill disturbances to quantify the balance control capacity of individuals with PD (Kuhman et al., 2020). The use of a treadmill as a tool to assess dynamic balance control standardizes test delivery compared to common neurological assessments of balance (i.e., the pull test and release from lean) (Lu et al., 2023; Jansen et al., 2025). Indeed, a recent study showed that variation between and within assessors in the execution of the balance assessment led to ≥ 2 points on a five-point rating scale in test outcomes for almost a third of participants (Jansen et al., 2025). Comparatively, in the same study, using a standardized treadmill protocol reduced variation in test outcomes to 8% (Jansen et al., 2025). In addition to standardizing test delivery, the current protocol removes the ceiling effect that standard neurological tests are subject to. Determining the largest acceleration that an individual can withstand while complying with the instructions of the task (i.e., avoid taking a step) challenges the balance control capacity of the PD nervous system. Further, our data suggests that increasing the step threshold to one level higher (i.e., a ST value ≥ 0.5 ms^–2^) is evidence of a true change in dynamic balance control. It should be noted that the current protocol encourages individuals to avoid stepping which is a natural response when recovering from a postural disturbance. Suppressing the natural stepping response can challenge response inhibition and reaction time consistency (Riccio et al., 2002).

Improved performance on the step threshold task might suggest that improved motor inhibition allowed individuals to regain stability without stepping. Step threshold performance correlated with executive function, providing evidence that medication-mediated changes in executive function impact balance control in patients with PD (Pullman et al., 1988; Beste et al., 2009). This includes the measure of within-person reaction time variability on the CPT-3, which also is conceptualized to reflect executive dysfunction (Del Bene et al., 2025). Within our data we observed a negative correlation with step threshold OFF medication and the CPT-3. This suggests that worse dynamic balance control is related to worse executive function. We also observed significant positive relationships between medication-mediated changes in step threshold and executive function. Our interpretation is that participants with larger medication-related improvements in their step threshold had lower thresholds in the OFF state and concomitantly higher scores on the CPT-3 (i.e., performance T > 60 is clinically meaningful). Reduction of corticostriatal dopaminergic inputs yields worse performance on response inhibition tasks in animal models and humans, thus it is reasonable to interpret reduced step thresholds OFF medication resulting from reduced response inhibition (Young et al., 2011). In the OFF-medication state, reduced corticostriatal dopamine could reduce the extent that individuals are able to suppress the stepping response, which is similar to previous studies that show that lower uptake of dopamine due to reduced number of receptors relates to functional motor outcomes (Nieoullon, 2002; Ghahremani et al., 2012). On a stop-signal task, another measure of response inhibition, there was a significant positive correlation between stop signal reaction time and dopamine release from several cortical regions involved in inhibitory control (Albrecht et al., 2014). While we offer these potential mechanisms, we also acknowledge that a mechanistic explanation is beyond the scope of the current investigation. In the current study, we show an improvement in step threshold capacity of 1 full level, on average after taking dopaminergic medication which correlated with performance on tests that assess aspects of executive function.

While the current study utilized equipment that is not common in most clinical settings there are alternative approaches to perform this protocol. A recent study used a waist-mounted spring scale to assess stepping thresholds in individuals with diabetes (Rosenblatt et al., 2021). In this task, increasing body lean created incremental loads as a percentage of body weight measured via the scale to standardize the task. Individuals were released without warning and instructed to try to avoid stepping (Rosenblatt et al., 2021). Thus, it is possible to implement this task in a clinical environment.

## 5 Limitations

Several limitations should be considered when interpreting our results. We did not randomize the performance of the OFF vs. ON medication trials, and the researchers were not blinded to the participants’ medication status. Since the participant needed to refrain from taking medication for at least 12 h before the OFF testing, the presentation of the step threshold task could not be randomized unless we tested on different days, however, outcomes of this task are objective, and the instructions provided to participants are standardized. We also did not randomize the presentation of perturbation trials to participants. However, this is not feasible for this type of protocol without knowing what each person’s step threshold is *a priori*. Regardless, individuals are provided opportunities to adapt recovery responses within four tries for each disturbance level they step at. Thus, even without randomization, we feel the protocol captures participants’ true step threshold. The interpretation that our results are due to improved functioning of executive function was not confirmed with objective neural data. Future studies incorporating neural data acquisition would help further establish the involvement of executive function at improving step threshold performance. Another limitation is that the difference in generation of postural disturbance occurring at the feet or shoulder level may alter the neurobiological recovery response. Indeed, a recent study did show differences in recovery response between the two modalities of balance disturbance (Lu et al., 2023). Regardless, we did show a significant correlation between the step threshold task and the pull test. One more limitation is that the large range in dopaminergic medication dosage and responsiveness to those medications could contribute to individual differences in the changes to step threshold.

## 6 Conclusion

The step threshold assessment is a valid protocol to evaluate dynamic balance control for individuals with PD. Measuring medication-mediated changes in dynamic balance control is important in patients with advanced PD, in whom disease progression and executive function can play roles in functional mobility and risk for falls. The step threshold is a capacity measure that is scalable and allows adaptation to the task. Future studies should establish stratified normalized ranges for step thresholds specifying the clinical significance for individuals with different disease severity or different disease populations prone to high fall risks. Overall, perturbation protocols can serve as key outcome variables to assess interventions, evaluate treatment efficacy, and monitor disease progression.

## Data Availability

The raw data supporting the conclusions of this article will be made available by the authors, without undue reservation.
